# Use of Legumes and Yeast as Novel Dietary Protein Sources in Extruded Canine Diets

**DOI:** 10.3389/fvets.2021.667642

**Published:** 2021-06-04

**Authors:** Lauren M. Reilly, Fei He, Sandra L. Rodriguez-Zas, Bruce R. Southey, Jolene M. Hoke, Gary M. Davenport, Maria R. C. de Godoy

**Affiliations:** ^1^Department of Animal Sciences, University of Illinois, Urbana, IL, United States; ^2^ADM, Decatur, IL, United States

**Keywords:** dog, digestibility, legume, microbiota, pulse, yeast

## Abstract

The popularity of plant-based protein sources has increased as consumer demand for grain-free and novel protein sources increase. Minimal research has been conducted as regards to use of legumes and yeast and their effects on acceptability and digestibility in canine diets. The objective of this study was to evaluate macronutrient apparent total tract digestibility (ATTD), gastrointestinal tolerance, and fermentative end-products in extruded, canine diets. Five diets were formulated to be isocaloric and isonitrogenous with either garbanzo beans (GBD), green lentils (GLD), peanut flour (PFD), dried yeast (DYD), or poultry by-product meal (CON) as the primary protein sources. Ten adult, intact, female beagles (mean age: 4.2 ± 1.1 yr, mean weight: 11.9 ± 1.3 kg) were used in a replicated, 5 × 5 Latin square design with 14 d periods. Each experimental period consisted of 10 d of diet adaptation, followed by 4 d of total fecal and urine collection. A fasted, 5 ml blood sample was collected at the end of each period and analyzed for serum metabolites and complete blood count. Serum metabolites were within normal ranges and all dogs remained healthy throughout the study. Fecal quality, evaluated on a 5-point scale, was considered ideal. Macronutrient ATTD was similar among dietary treatments, with diets highly digestible (>80%). Total fecal branched-chain fatty acid concentrations were highest (*P* < 0.05) for DYD (23.4 μmol/g) than GLD (16.1 μmol/g) and PFD (16.0 μmol/g) but not different (*P* > 0.05) than other treatments. The plant-based protein treatments had greater (*P* < 0.05) total fecal short chain fatty acid (SCFA) concentrations (average 627.6 μmol/g) compared with CON (381.1 μmol/g). Fecal butyrate concentration was highest (*P* < 0.05) for DYD than all other dietary treatments (103.9 μmol/g vs. average 46.2 μmol/g). Fecal microbial communities showed *Firmicutes, Bacteroidetes, Fusobacteria*, and *Proteobacteria* as abundant phyla. There was greater β-diversity for dogs fed DYD which differed from all other diets in both weighted and unweighted UNIFRAC analyses. Inclusion of these novel, plant-based, protein sources showed no detrimental effects on nutrient digestibility or fecal characteristics and represent viable protein sources in canine diets that can produce beneficial shifts in fecal metabolites.

## Introduction

Recent trends in the pet food industry have centered on providing high-protein diets that contain novel protein sources. Legumes, pulses, and yeast, have been identified as alternatives to traditional protein sources (i.e., animal-derived protein) to satisfy consumer demand for non-traditional diets, such as grain-free, while simultaneously meeting the nutritional requirements of dogs.

The inclusion of legumes and pulses (crops in the *Leguminosae* family harvested for the dry grain) can negatively impact digestibility and fecal quality due to presence of anti-nutritional factors and oligosaccharides (1, 2). However, they are protein-rich ingredients that are easily incorporated into pet diets because of their low lipid content (3, 4). Yeast (*Saccharomyces cerevisiae*) products have shown beneficial health effects in several animal models including the modulatory effects of colonic microbiota in dogs (5–7).

Minimal research has been conducted as regards to the effects on acceptability, digestibility, and fecal characteristics of legumes and yeast when used as main protein sources in extruded canine diets. Therefore, the objectives of this study were to evaluate the effects of extruded canine diets containing green lentils (GLD), garbanzo beans (GBD), peanut flour (PFD), or dried yeast (DYD) on ATTD, gastrointestinal tolerance, fermentative end-products, and fecal microbiota populations. It was hypothesized that extruded diets containing legumes or yeast would result in similar macronutrient digestibility to poultry by-product meal (CON), as most of the anti-nutritional factors would be deactivated during thermal processing. Greater saccharolytic fermentation was expected in dogs fed the legume or yeast containing-diets due to their intrinsically greater dietary fiber content and composition.

## Materials and Methods

### Experimental Design

All animal care protocols used in this study were approved by the Institutional Animal Care and Use Committee at the University of Illinois at Urbana-Champaign. All methods were performed in accordance with the United States Public Health Service Policy on Humane Care and Use of Laboratory Animals.

Ten adult, intact, female Beagles (average age: 5.0 ± 1.2 yr, average weight: 11.9 ± 1.3 kg, average body condition score: 5.9 on a 9-point scale) were used in a replicated 5 × 5 Latin square design. Each 14 d experimental period consisted of a 10 d diet adaptation phase, followed by a 4 d total fecal and urine collection phase. On day 14 of each experimental period, a 5 ml, fasted, blood sample was collected from all dogs for a serum chemistry and complete blood count (**CBC**) analyses conducted at the University of Illinois Veterinary Medicine Diagnostics Laboratory (Urbana, IL). The dogs were housed in a temperature-controlled room at the Veterinary Medicine Basic Sciences Building with a 14 h light/10 h dark schedule. The dogs were individually housed in kennels (1.2 m wide by 1.8 m long) during the diet adaptation phases which permitted nose to nose contact with dogs in adjacent runs and visual contact with all dogs in the room. During collection phases, the dogs were individually housed in metabolic crates. The dogs were fed twice daily (8:00 and 16:00) with *ad libitum* access to water throughout the study.

Dogs were randomly assigned to one of five diets formulated with either garbanzo beans (GBD), green lentils (GLD), peanut flour (PFD), a dried yeast product (DYD), or poultry by-product meal (CON) as the primary protein source (Table 1). The legumes and yeast were included at the expense of poultry by-product meal and rice to provide test diets with similar nutrient composition and energy content. All diets were formulated to be complete and balanced according to AAFCO (8) recommended values for adult dogs at maintenance. Diets were extruded by Wenger Manufacturing (Wenger Manufacturing, Inc., Sabetha, KS). Extrusion processing parameters (Supplementary Table 1) were adjusted as needed to ensure uniformity of the final product characteristics (e.g., density, texture, and kibble size). Food intake was individually calculated to maintain body weight based on metabolizable energy requirements. Any food refusals were measured after each meal throughout the duration of the study. Body weight and body condition were measured weekly and food intake was adjusted accordingly during the adaptation phase to maintain body weight, if necessary.

**Table 1:** Ingredient composition of canine diets containing legumes or yeast.

	**Dietary treatment**				
**Ingredient**	**Control**	**Garbanzo bean**	**Green lentils**	**Peanut flour**	**Dried yeast**
Garbanzo bean	–	43.56	–	–	–
Green lentil	–	–	44.65	–	–
Peanut flour	–	–	–	28.08	–
Dried yeast	–	–	–	–	29.88
Poultry by-product meal	33.5	22.26	19.15	11.22	10.00
Rice	42.96	10.00	10.00	38.46	33.87
Poultry fat	8.47	8.74	10.14	4.43	8.26
Corn	10.00	10.00	10.00	10.00	10.00
Dried beet pulp	2.50	2.50	2.50	2.50	2.50
Palatant	1.00	1.00	1.00	1.00	1.00
Ca carbonate	0.78	0.66	0.57	1.51	1.89
Dical. phosphate	–	0.49	1.20	2.00	1.80
Salt	0.30	0.30	0.30	0.30	0.30
Vitamin premix^1^	0.18	0.18	0.18	0.18	0.18
Mineral premix^2^	0.18	0.18	0.18	0.18	0.18
Choline chloride	0.12	0.12	0.12	0.12	0.12
BHT (antioxidant)	0.02	0.02	0.02	0.02	0.02

1 *Provided per kg diet: 17.4 mg manganese (MnSO_4_), 284.3 mg iron (FeSO_4_), 17.2 mg copper (CuSO_4_), 2.2 mg cobalt (CoSO_4_), 166.3 mg zinc (ZnSO_4_), 7.5 mg iodine (KI), and 0.2 mg selenium (Na2SeO_3_)*.

2 *Provided per kg diet: 10.8 mg copper (CuSO_4_), 0.36 mg selenium (Na_2_SeO_3_), 150 mg zinc (ZnSO_4_, ZnO), 2,562.8 IU vitamin A, 254 IU vitamin D3, 32.1 IU vitamin E*.

### Sample Collection and Preparation

A fresh fecal sample was collected from each dog within 15 min of defecation and analyzed for pH, dry matter (DM), short-chain fatty acids (SCFA), branched-chain fatty acids (BCFA), ammonia, phenols, indoles, and microbiota. All fecal samples were assessed for fecal quality using 5-point scoring system: 1 = hard, dry pellets; 2 = hard formed, remains firm and soft, 3 = soft, formed and moist stool; 4 = soft, unformed stool; or 5 = watery, liquid that can be poured. Dry matter was analyzed in 2 g duplicates and dried for 48 h in a 105°C forced-air oven. For analysis of BCFA, SCFA, and ammonia, 5 g of feces were collected in a 30 ml Nalgene bottle containing 5 ml of 2 N hydrochloric acid. Phenols/indoles were collected by weighing 2 g of feces into duplicate plastic tubes and covered in Parafilm. Short-chain fatty acid and phenol/indole samples were stored at −20°C until analysis. Fecal sample allocated for microbiota were stored in 2 ml cryovials and stored at −80°C until analysis.

Total feces and urine were collected simultaneously during the 4 d collection phase of the study. All fecal samples were weighed, quality scored, and stored at −20°C until analyzed to determine macronutrient apparent total tract digestibility (ATTD). Total urine was collected into Nalgene bottles containing 10 ml of 2 N hydrochloric acid and weighed. A 25% subsample of the total weight was collected and stored at −20°C until analysis.

A 5 ml, fasted, blood sample was collected via jugular venipuncture from all dogs as a health check at the end of each experimental period. Serum metabolites were analyzed using 4 ml of blood collected in a serum separator vacutainer tube and a CBC was analyzed using the remaining 1 ml of blood collected in an EDTA vacutainer tube (Becton, Dickinson and Company, Franklin Lakes, NJ).

### Maillard Reaction Product Analysis

A sample of each diet was analyzed for the presence of Maillard reaction products (MRP). The analyzed MRP were hydroxymethylfurfural (HMF), furosine (FS), carboxymethyllysine (CML), and fructoselysine (FL). Reactive lysine content of the dietary treatments were calculated using the furosine procedure (9).

The samples were analyzed for HMF using the HPLC procedure with modifications (10). Dried sample (100 mg) was homogenized for 30 min with 1.3 ml of 1.2% (w/v) glacial acetic acid solution in water and 50 μl of Carraz I and 50 μl of Carraz II reagents (Carrez Clarification Kit, Sigma-Aldrich, St. Louis, MO). The mixture then was centrifuged (model 5416C Eppendorf Centrifuge, Brinkman Instruments, Inc., Westbury, NY) at a rate of 10,000 x *g* for 30 min. The supernatant was filtered through 0.2 μm PTFE filter. An isocratic HPLC system was used with Alliance 2695 separation module (Waters Corporation, Milford, MA), an Inertsil ODS-3 column (25 cm × 0.46 cm i.d. × 5μm df; MetaChem Technologies, Inc., Torrance, CA), and a 1050 Diode Array Detector (DAD, Agilent Technologies, Inc., Palo Alto, CA). HPLC grade water and methanol, added in a ratio of 90:10 (v/v), was used as a mobile phase at 1 ml/min flow rate for separations. The wavelength for HMF was detected at 284 nm using a UV detector.

Samples were analyzed for FS and CML using gas chromatography-mass spectrometry (11). The defatting step was modified by adding 50 mg of the dried sample and 5 ml pentane to a 15 ml screw cap, glass tube with a PTFE-lined cap. The samples then were vortexed for 5 min, centrifuged to separate the particulate, and the excess pentane was removed. A 50 μl internal standard solution, composed of 1.15 mg/ml of cycloleucine in water, was added. The defatted sample then was hydrolyzed and derivatized (11). Concentration of FL was calculated from FS (12).

### Chemical Analyses

Fecal samples were composited by dog and experimental period and dried in a 57°C forced-air oven. Diet and fecal samples were ground through a 2 mm screen using a Wiley mill (model 4, Thomas Scientific, Swedesboro, NJ) and were analyzed for DM, ash, organic matter (OM), acid hydrolyzed fat (AHF), crude protein (CP), gross energy (GE), and total dietary fiber (TDF). Dry matter, ash, and OM were determined according to AOAC (13) methods 934.01 and 942.05. Total nitrogen values were determined according to AOAC (13) method 992.15 with CP calculated from Leco (TruMac N, Leco Corporation, St. Joseph, MI). Acid hydrolyzed fat, used to measure total fat content (14, 15). Gross energy was analyzed with bomb calorimetry (Model 6200, Parr Instruments Co., Moline, IL). Total dietary fiber was analyzed (16).

Fecal concentrations of SCFA and BCFA were measured using gas chromatography (17). Fecal ammonia concentrations were determined (18). Fecal phenol and indole concentrations were analyzed through gas chromatography (19).

### Anti-nutritional Factors and Oligosaccharides

Processing samples were collected for all diets throughout the extrusion process. Samples were taken of the base mix, after the preconditioner, after the extruder, and the coated final diet at multiple time-points. Processing samples were analyzed for trypsin inhibitors according to AACC (14) methods 22–40 s and urease activity according to AACC (14) methods 22–90. Free sugar profiles of substrates were determined (20, 21).

### DNA Extraction, Amplification, Sequencing, and Bioinformatics

Total DNA was extracted from fresh fecal samples using a Mo-Bio PowerSoil kit (MO BIO Laboratories, Inc., Carlsbad, CA). Quantification of DNA concentration was completed using a Qubit® 2.0 Fluorometer (Life technologies, Grand Island, NY). A Fluidigm Access Array (Fluidigm Corporation, South San Francisco, CA), in combination with Roche High Fidelity Fast Start Kit (Roche, Indianapolis, IN), were used for amplification of the 16S rRNA gene. The primers 515F (5′-GTGCCAGCMGCCGCGGTAA-3′) and 806R (5′-GGACTACHVGGGTWTCTAAT-3′), targeting a 292 bp-fragment of V4 region, were used for amplification (primers synthesized by IDT Corp., Coralville, IA) (22). Fluidigm specific primer, forward and reverse tags, were added in accordance with the Fluidigm protocol. The quality of amplicons' regions and sizes were confirmed by Fragment Analyzer (Advanced Analytics, Ames, IA). A DNA pool was generated through the combination of equimolar amounts of the amplicons from each sample. The pooled samples were selected by size on a 2% agarose E-gel (Life Technologies, Grand Island, NY) and extracted using a Qiagen gel purification kit (Qiagen, Valencia, CA). The pooled, size-selected, and cleaned products were then analyzed on an Agilent Bioanalyzer to confirm appropriate profile and mean size. The Roy J. Carver Biotechnology Center at the University of Illinois performed Illumina sequencing on a MiSeq using v3 reagents (Illumina Inc., San Diego, CA). A FASTX-Toolkit (version 0.0.14) removed the Fluidigm tags. Analysis of sequences was completed using QIIME 2.0 (22) and DADA2 (version 1.14) (23). The high quality (quality value ≥ 20) sequence data, derived from the sequencing process, were de-multiplexed. An opened-reference OTU clustered the sequences into operational taxonomic units (OTU), choosing against the SILVA 138 reference OTU database with a 97% similarity threshold (24). The OTUs observed fewer than 2 times (singletons), as well as OTUs with <0.01% of the total observation were discarded. An average of 56,012 reads were obtained, with a total of 2,800,636 reads. The number of reads ranged from 37,603 to 79,027 per sample. Rarefaction curves based on observed species, Chao1, and the plateaus observed in the phylogenetic distance whole tree measures suggest sufficient sequencing depth. To analyze for diversity and species richness, the dataset was rarified to 37,600 reads. Weighted and unweighted unique fraction metric (UniFrac) distances were performed by principal coordinates analysis (PCoA). This measured the phylogenetic distance between sets of taxa in a phylogenetic tree as a fraction of the branch length of the tree, based on the 97% OTU composition and abundance matrix (25).

### Statistical Analyses

All data were analyzed in SAS (SAS Institute, Inc., version 9.4, Cary, NC) using the MIXED models procedure with the exception of fecal score data, which were analyzed using the GLIMMIX procedure. The model was run with a fixed effect of diet and a random effect of dog. The differences among treatments were reported using a Fisher-protected least significance test with a Tukey adjustment to control for the Type 1 experiment-wise error. Means were considered to be statistically significant using a probability of *P* < 0.05. The reported standard errors of the means (SEM) were determined from the MIXED models procedure in SAS. Statistical analysis could not be performed on the MRP, anti-nutritional factor, or oligosaccharide data because the procedures were only performed using technical replicates.

## Results

### Diet Proximate Analysis, Food Intake, and Fecal Characteristics

Ingredient composition (Table 1) of all five diets was targeted to be similar with the exception of the test ingredient. The percent inclusion of the test ingredients varied in order for the diets to have similar macronutrient composition (Table 2). Dry matter content of the diets ranged from 91.8 to 93.9%. Macronutrient composition of the dietary treatments is reported on a dry matter basis (DMB). Control had the highest CP content (31.2%) compared with the other dietary treatments, which ranged from 26.6 (PFD) to 29.1% (DYD).

**Table 2:** Analyzed chemical composition and energy content of canine diets containing legumes or yeast.

	**Dietary treatment**^**1**^				
**Item**	**CON**	**GBD**	**GLD**	**PFD**	**DYD**
Dry matter (%)	91.8	92.5	92.2	93.9	91.8
	**Dry matter basis**				
Crude protein (%)	31.2	26.9	27.4	26.6	29.1
Acid hydrolyzed fat (%)	15.9	17.3	14.5	15.1	15.6
Total dietary fiber (%)	11.2	11.9	11.2	10.5	17.7
Soluble (%)	5.6	3.9	4.2	4.6	6.8
Insoluble (%)	5.6	8.1	7.0	5.9	10.9
Ash (%)	7.2	7.0	7.1	7.0	7.6
Gross energy (kcal/g)	5.1	5.0	5.0	5.1	5.1

1 *CON, Poultry by-product meal control; GBD, Garbanzo bean; GLD, Green lentil; PFD, Peanut flour; DYD, Dried yeast*.

Food intake (g/d, DMB) was significantly lower (*P* < 0.05) for GLD, with an intake of 169.1 g/d, compared with CON, with an intake of 175.6 g/d (Table 3). Other dietary treatments were not different from each other (*P* > 0.05).

**Table 3:** Food intake, fecal scores, and fecal output of dogs fed diets containing legumes or yeast as the primary protein source.

	**Dietary treatment**^**1**^					
**Item**	**CON**	**GBD**	**GLD**	**PFD**	**DYD**	**SEM^2^**
Food intake (g/d, as-is)	191.2^a^	186.9^ab^	183.3^b^	185.1^b^	186.1^ab^	4.235
Food intake (g/d, DMB^3^)	175.6^a^	172.9^ab^	169.1^b^	173.7^ab^	170.8^ab^	3.918
Fecal score	2.2	2.5	2.8	2.5	2.4	0.231
Fecal output (g/d, as-is)	58.2^c^	89.6^ab^	93.7^a^	73.2^bc^	93.6^a^	6.021
Fecal output (g/d, DMB^3^)	24.1^c^	28.1^bc^	30.8^ab^	24.8^c^	33.6^a^	1.541
Urine output (ml/d)	161.8	172.7	152.1	159.8	135.9	16.707

1 *CON, Poultry by-product meal control; GBD, Garbanzo bean; GLD, Green lentil; PFD, Peanut flour; DYD, dried yeast*.

2 *SEM, Standard error of the mean*.

3 *DMB, Dry matter basis*.

a−c *Means within a row with different superscripts are different (P < 0.05)*.

Fecal output (g/d, DMB) was highest (*P* < 0.05) for DYD at 33.6 g/d, but not different (*P* > 0.05) from GLD which was 30.8 g/d (Table 3). Control had the lowest (*P* < 0.05) fecal output at 24.1 g/d but did not differ (*P* > 0.05) from GBD (28.1 g/d) or PFD (24.8 g/d). Fecal output on an as-is basis followed the same pattern with DYD as the highest (*P* < 0.05) at 93.6 g/d and CON as the lowest (*P* < 0.05) at 58.2 g/d. Despite the differences in fecal output, no significant differences (*P* > 0.05) were observed in fecal scores among all treatments (Table 3). All fecal scores were within the ideal range of 2–3 with an average value of 2.5. No significant differences (*P* > 0.05) were observed in total urine output (Table 3) among treatments.

### Maillard Reaction Products, Anti-nutritional Factors, and Oligosaccharides

**Figure 1:**
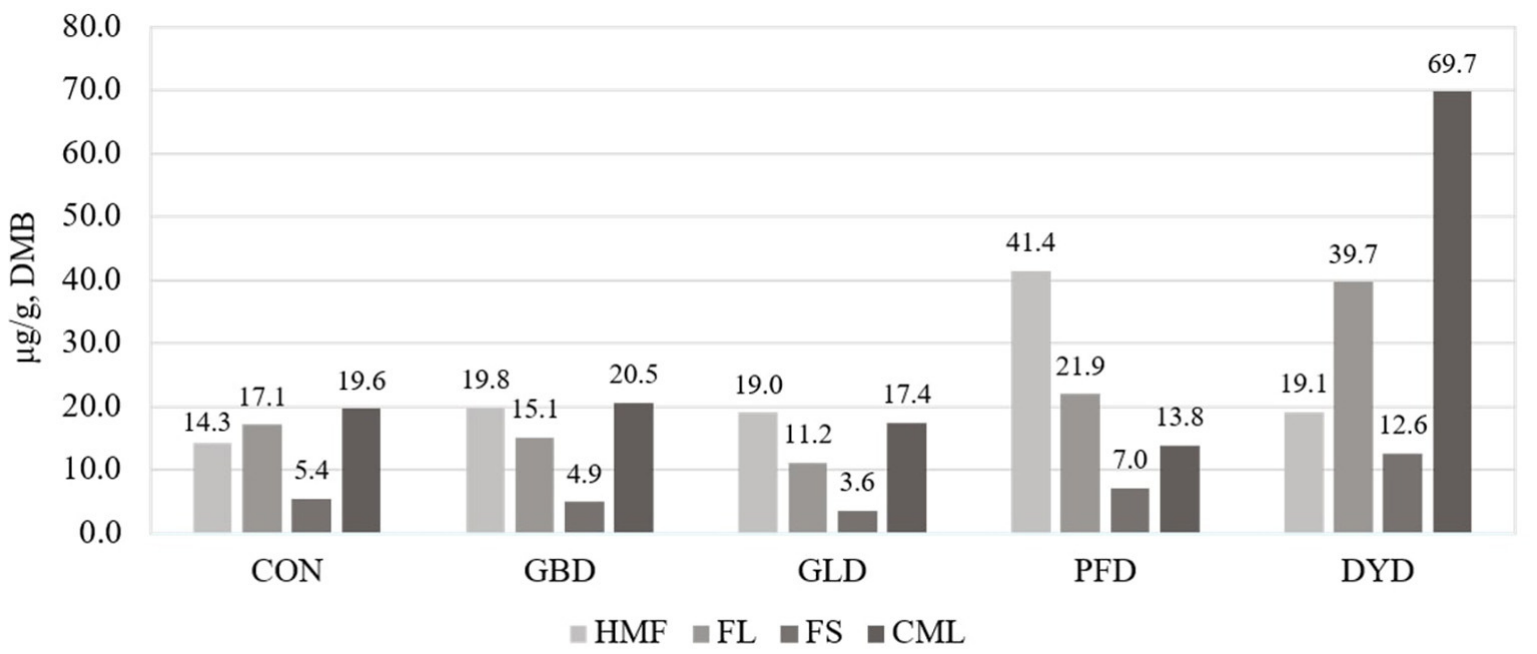
Presence of Maillard reaction products (MRPs) in canine diets containing legumes or yeast as the primary protein source. DMB, Dry matter basis; CON, Poultry by-product meal control; GBD, Garbanzo bean; GLD, Green lentil; PFD, Peanut flour; DYD, Dried yeast; HMF, Hydroxymethylfurfural; FL, Fructoselysine; FS, Furosine; CML, Carboxymethyllysine. Statistical analysis could not performed due to use of technical replicates.

**Figure 2:**
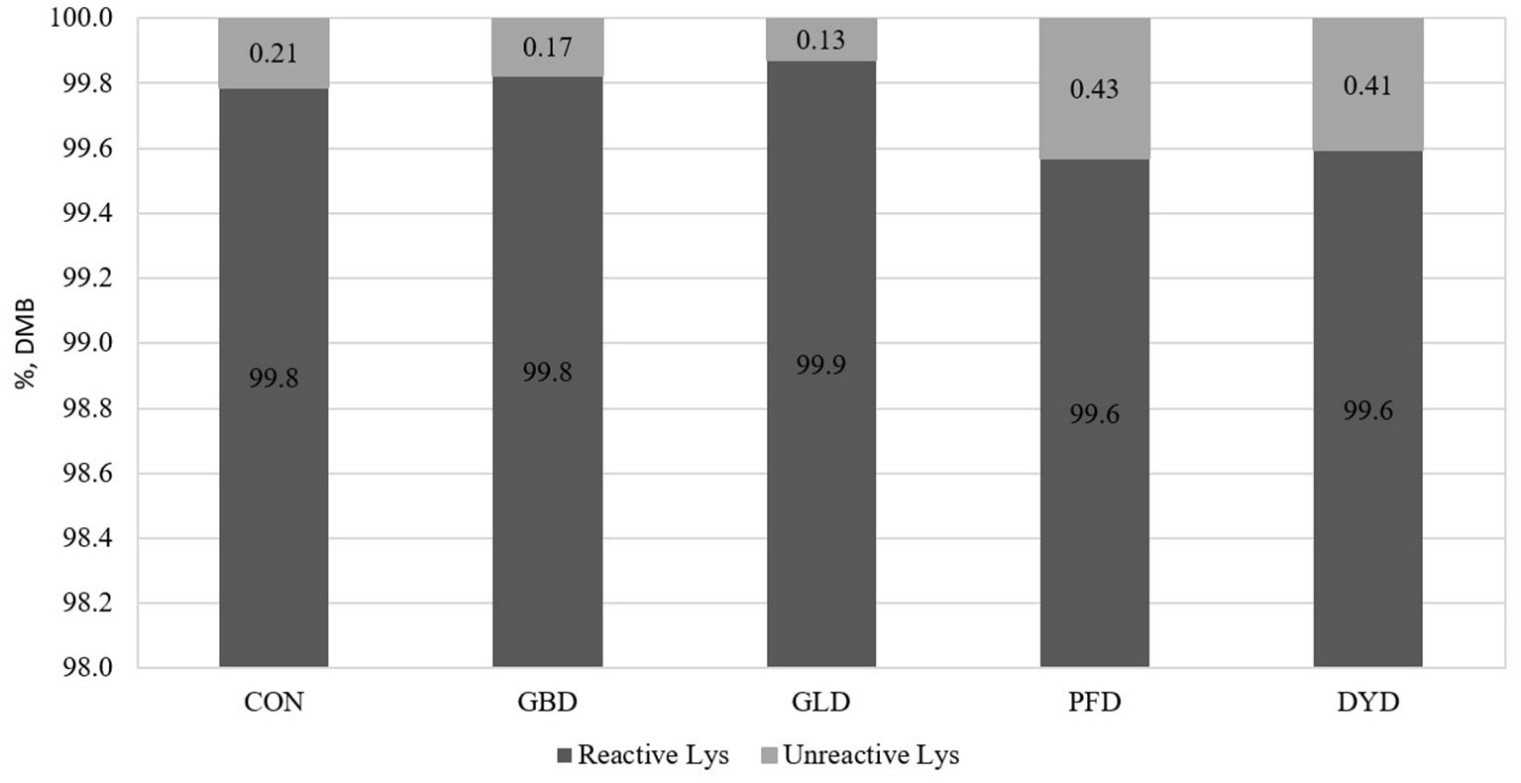
Reactive lysine content in canine diets containing legumes or yeast as the primary protein source. DMB, Dry matter basis; CON, Poultry by-product meal control; GBD, Garbanzo bean; GLD, Green lentil; PFD, Peanut flour; DYD, Dried yeast.

The concentration of MRP (Figure 1) present in each of the dietary treatments used in this study were measured. The concentration of hydroxymethylfurfural (HMF) was highest in PFD (41.4 μg/g, DMB) compared with the other treatments, which had an average HMF concentration of 18.1 μg/g, DMB. In the current study, DYD had the highest concentrations of fructoselysine (FL), furosine (FS), and carboxymethyllysine (CML) at 39.7, 12.6, and 69.7 μg/g, respectively. For FL, the remaining dietary treatments ranged from 11.2 μg/g (GLD) to 21.9 μg/g (PFD). The range of FS concentration for the remaining dietary treatments was narrower from 3.6 μg/g (GLD) to 7.0 μg/g (PFD). Lastly, for CML, the remaining diets ranged from 13.8 (PFD) to 20.5 μg/g (GBD). The dietary treatments were determined to have minimal heat damage (Figure 2), with reactive lysine contents ranging from 99.6% (DMB) in PFD and DYD to 99.9% in GLD.

Anti-nutritional factor content for the dietary treatments at various stages of processing (Table 4) showed trypsin inhibitor activity present in GBD and GLD at all stages of processing until the final diet. The GBD base mix had trypsin inhibitor activity measured at 4,385.0 TIU/g. After the preconditioner, the activity was similar at 4,486.9 TIU/g, and then decreased after the extruder to 1,182.8 TIU/g (DMB). There was no urease activity detectable for any of the diets at any processing stage.

**Table 4:** Anti-nutritional factors and oligosaccharide content of legume-based canine diets at various processing stages.

	**Anti-nutritional factors (DMB**^**1**^**)**		**Oligosaccharides (%, DMB**^**1**^**)**		
**Diet stage of processing^**2**^**	**Trypsin inhibitor (TIU/g)**	**Urease activity (Δ pH units)**	**Raffinose**	**Stachyose**	**Verbascose**
**Base mix**					
CON	0	0	0.04	0.03	0
GBD	4,385.0	0	0.27	0.61	0.01
GLD	731.3	0	0.14	1.15	0.39
**After preconditioner**					
CON	0	0	0.03	0.05	0
GBD	4,486.9	0	0.28	0.26	0.01
GLD	1,533.7	0	0.15	0.56	0.08
**After extruder**					
CON	0	0	0.05	0.04	0
GBD	1,182.8	0	0.24	0.66	0.03
GLD	495.9	0	0.17	1.22	0.31
**Final diet**					
CON	0	0	0.03	0.03	0.01
GBD	0	0	0.27	0.72	0.02
GLD	0	0	0.15	1.17	0.43

1 *DMB, Dry matter basis*.

2 *CON, Poultry by-product meal control; GBD, Garbanzo bean diet; GLD, Green lentil diet*.

Oligosaccharide content of the diets was measured at various processing stages (Table 4). Raffinose content was numerically higher for GBD at all processing stages, ranging from 0.24 to 0.28% (DMB). Stachyose content was higher for GLD at each processing stage, ranging from 0.56% (after preconditioner) to 1.22% (after extruder). Similarly, verbascose content was highest in GLD at each processing stage (0.39% base mix, 0.08% after preconditioner, 0.31% after extruder, and 0.43% in the final diet) in contrast with other dietary treatments.

### Apparent Total Tract Macronutrient and Energy Digestibility

All diets were well-digested with macronutrient ATTD at or above 80% (Table 5). In terms of DM, OM, CP, and AHF, PFD and CON diets consistently had the highest (*P* < 0.05) ATTD. Dogs fed DYD consistently had the lowest (*P* < 0.05) ATTD for DM (80.1%), OM (84.3%), CP (83.7%), and AHF (87.9%). The TDF digestibility of the diets did not differ (*P* > 0.05), with the exception of GLD which was significantly lower (*P* < 0.05) at 26.7% than all other dietary treatments. Digestible energy content (Table 5) were highest for CON (4.7 kcal/g) and lowest (*P* < 0.05) for DYD with values of 4.3 kcal/g (DMB) but was not different (*P* > 0.05) from GLD with values of 4.4 kcal/g. Metabolizable energy content (Table 5) followed the same pattern as DE, with dogs fed CON having the highest (*P* < 0.05) ME of 4.4 kcal/g and dogs fed DYD and GLD having the lowest (*P* < 0.05) at 4.0 and 4.1 kcal/g, respectively.

**Table 5:** Apparent total tract macronutrient and energy digestibility of dogs fed diets containing legumes or yeast as the primary protein source.

	**Dietary treatment**^**1**^					
**Nutrient digestibility**	**CON**	**GBD**	**GLD**	**PFD**	**DYD**	**SEM^**2**^**
Dry matter (%)	86.3^a^	83.7^ab^	82.1^bc^	85.6^a^	80.1^c^	0.793
Organic matter (%)	91.2^a^	87.5^b^	85.8^bc^	90.3^a^	84.3^c^	0.617
Crude protein (%)	86.6^a^	83.2^bc^	81.5^c^	85.0^ab^	83.7^abc^	0.863
Acid hydrolyzed fat (%)	94.7^a^	94.2^ab^	94.1^b^	95.5^a^	87.9^c^	0.327
Total dietary fiber (%)	50.8^a^	44.2^a^	26.7^b^	52.9^a^	42.2^a^	3.132
Digestible energy (kcal/g)	4.69^a^	4.46^bc^	4.37^cd^	4.49^b^	4.29^d^	0.029
Metabolizable energy (kcal/g)	4.44^a^	4.26^b^	4.13^bc^	4.27^b^	4.04^c^	0.041

1 *CON, Poultry by-product meal control; GBD, Garbanzo bean; GLD, Green lentil; PFD, Peanut flour; DYD, dried yeast*.

2 *SEM, Standard error of the mean*.

a−d *Means within a row with different superscripts are different (P < 0.05)*.

### Fecal Fermentative End-Products and Serum Chemistry

Total fecal concentrations of SCFA (μmol/g, DMB) were lowest (*P* < 0.05) for CON at 381.1 μmol/g (Table 6). Total fecal SCFA concentrations in the other dietary treatments were not significantly different from each other (*P* > 0.05) with GBD being the highest at 711.0 μmol/g. Acetate and propionate concentrations were highest (*P* < 0.05) for GBD at 459.5 μmol/g and 207.2 μmol/g, respectively. At 103.9 μmol/g, DYD had the highest (*P* < 0.05) butyrate concentration. This is more than twice the butyrate concentrations measured in the other dietary treatments, which were not significantly different from each other (*P* > 0.05) with an average of 46.2 μmol/g.

**Table 6:** Fecal fermentative end-product concentrations of dogs fed diets containing legumes or yeast as the primary protein source.

	**Dietary treatment**^**1**^					
**Item (DMB^**2**^)**	**CON**	**GBD**	**GLD**	**PFD**	**DYD**	**SEM^**3**^**
Fecal pH	6.57^a^	6.05^ab^	6.03^b^	6.37^ab^	6.05^ab^	0.135
Ammonia (mg/g)	2.3^a^	1.9^ab^	1.9^ab^	1.6^b^	2.1^ab^	0.124
**SCFA**^**4**^ **(μmol/g)**						
Acetate	221.1^c^	459.5^a^	368.1^b^	361.6^b^	349.7^b^	19.803
Propionate	114.8^b^	207.2^a^	198.5^ab^	172.3^ab^	150.4^ab^	21.637
Butyrate	45.2^b^	44.4^b^	49.7^b^	45.4^b^	103.9^a^	7.604
Total SCFA	381.1^b^	711.0^a^	616.2^a^	579.3^a^	604.0^a^	37.575
**BCFA**^**4**^ **(μmol/g)**						
Isobutyrate	7.8	6.5	5.6	6.3	7.4	0.653
Isovalerate	11.3^ab^	8.3^b^	8.6^ab^	8.6^ab^	12.3^a^	0.931
Valerate	1.0^b^	1.8^ab^	2.0^ab^	1.1^b^	3.6^a^	0.469
Total BCFA	20.1^ab^	16.7^ab^	16.1^b^	16.0^b^	23.4^a^	1.659
**Phenols/indoles (μmol/g)**						
Phenols	0.5^a^	0.5^a^	0.2^b^	0.5^a^	0.3^b^	0.101
Indoles	1.7^a^	0.9^b^	1.1^b^	1.1^b^	1.6^a^	0.121
Total Phenol/Indoles	2.2^a^	1.5^bc^	1.2^c^	1.7^bc^	1.9^b^	0.158

1 *CON, Poultry by-product meal control; GBD, Garbanzo bean; GLD, Green lentil; PFD, Peanut flour; DYD, dried yeast*.

2 *DMB, Dry matter basis; SEM, Standard error of the mean*.

3 *SEM, Standard error of the mean*.

4 *SCFA, Short chain fatty acids; BCFA, Branched chain fatty acids*.

a−c *Means within a row with different superscript letters are different (P < 0.05)*.

Total fecal BCFA concentrations (μmol/g, DMB) were highest (*P* < 0.05) for DYD at 23.4 μmol/g and lowest (*P* < 0.05) for GLD and PFD at 16.1 μmol/g and 16.0 μmol/g, respectively (Table 6). No significant differences were observed in isobutyrate concentrations among the dietary treatments. Isovalerate concentrations were higher (*P* < 0.05) for DYD at 12.3 μmol/g than GBD at 8.3 μmol/g. Valerate concentrations were also higher (*P* < 0.05) for DYD at 3.6 μmol/g than PFD (1.1 μmol/g) and CON (1.0 μmol/g).

Total fecal phenol and indole concentrations (μmol/g, DMB) were highest (*P* < 0.05) in dogs fed CON at 2.21 μmol/g (Table 6). Dogs fed GLD had the lowest (*P* < 0.05) total phenol and indole concentration with a value of 1.18 μmol/g. Phenols were lowest (*P* < 0.05) for GLD (0.2 μmol/g) and DYD (0.3 μmol/g) and highest (*P* < 0.05) for PFD, GBD and CON, both of which had a phenol concentration of 0.5 μmol/g. Indoles were highest (*P* < 0.05) in DYD and CON with values of 1.6 μmol/g and 1.7 μmol/g, respectively. The ammonia concentration (Table 6) was lower (*P* < 0.05) for PFD (1.6 mg/g) than CON (2.3 mg/g), with the rest of the treatments not different (*P* > 0.05) from each other.

Serum chemistry and a CBC were analyzed throughout the study to monitor health of the dogs and to ensure dietary treatments did not cause any negative health effects. All serum metabolites (Table 7) were within normal ranges for all dietary treatments. Additionally, the analyzed CBC components were determined to be normal for healthy adult dogs (data not shown).

**Table 7:** Serum metabolites of dogs fed diets containing legumes or yeast as primary protein sources.

		**Dietary treatment**^**1**^					
**Item**	**Reference range^**2**^**	**CON**	**GBD**	**GLD**	**PFD**	**DYD**	**SEM^**3**^**
Creatinine (mg/dL)	0.5–1.5	0.6	0.6	0.5	0.6	0.5	0.022
BUN (mg/dL)^3^	6.0–30.0	12.8	11.1	11.4	12.2	13.3	0.496
Total protein (g/dL)	5.1–7.0	6.1	5.9	5.9	5.8	6.2	0.125
Albumin (g/dL)	2.5–3.8	3.3	3.3	3.3	3.4	3.3	0.064
Globulin (g/dL)	2.7–4.4	2.7	2.6	2.6	2.6	2.7	0.097
Ca (mg/dL)	7.6–11.4	10.4	10.4	10.4	10.4	10.6	0.087
P (mg/dL)	2.7–5.2	4.3	4.4	4.8	4.3	4.2	0.146
Na (mmol/L)	141–152	144.6	144	144.5	145.1	144.9	0.464
K (mmol/L)	3.9–5.5	4.3	4.4	4.4	4.4	4.3	0.076
Na:K ratio	28–36	34.0	33.0	32.8	33.2	33.6	0.639
Cl (mmol/L)	107–118	109.7	109.9	109.5	109.9	109.9	0.672
Glucose (mg/dL)	68–126	97.0	95.7	91.1	96.0	95.3	2.511
Total Bilirubin (mg/dL)	0.1–0.3	0.2	0.2	0.2	0.2	0.2	0.011
Cholesterol (mg/dL)	129–297	235.9	237	216.2	226.8	213.8	13.741
Triglycerides (mg/dL)	32–154	76.0	72.2	73.5	84.4	67.0	7.093
Bicarbonate (mmol/L)	16–24	22.0	22.0	22.5	23.0	21.9	0.409

1 *CON, Poultry by-product meal control; GBD, Garbanzo bean; GLD, Green lentil; PFD, Peanut flour; DYD, dried yeast*.

2 *Reference ranges were provided by the University of Illinois Veterinary Diagnostics Laboratory*.

3 *SEM, Standard error of the mean; BUN, Blood urea nitrogen*.

### Fecal Microbiota

The microbial composition at the phylum level is shown in Table 8. The most abundant phyla included Firmicutes (ranging from 47% of the sequences for dogs fed PFD to 62% for dogs fed GBD), Bacteroidetes (16% for DYD to 25% PFD), and Fusobacteria (13% for GBD to 21% for CON). Proteobacteria corresponded to 7% or less of the sequences among dietary treatments. Actinobacteria comprised ~1% of the sequences overall, while Deferribacteres and Epsilonbacteraeota corresponded to <1% of the total sequences among dietary treaments.

**Table 8:** Relative abundance of bacterial phyla and families of dogs fed diets containing legumes or yeast as primary protein sources.

		**Dietary treatment**^**1**^					
**Phylum**	**Family**	**CON**	**GBD**	**GLD**	**PFD**	**DYD**	**SEM^**2**^**
Actinobacteria		0.9^ab^	1.9^a^	1.5^ab^	0.6^b^	0.9^ab^	0.308
	*Bifidobacteriaceae*	0.5^ab^	1.1^ab^	1.3^a^	0.3^b^	0.7^ab^	0.308
	*Coriobacteriaceae*	0.4^ab^	0.8^a^	0.2^b^	0.4^ab^	0.2^b^	0.133
Bacteroidetes		22.8^ab^	17.2^b^	20.2^ab^	25.4^a^	16.1^b^	1.988
	*Bacteriodaceae*	14.1	12.2	13.9	17.0	12.3	1.473
	*Prevotellaceae*	7.5^a^	4.7^b^	5.5^ab^	7.6^a^	3.8^b^	0.981
Firmicutes		48.2^b^	62.0^a^	55.3^ab^	46.7^b^	61.4^a^	3.298
	*Clostridiaceae*	0.2^b^	0.2^b^	0.1^b^	0.4^b^	3.5^a^	0.506
	*Lachnospiraceae*	18.9	16.6	14.6	18.1	15.1	1.718
	*Peptococcaceae*	0.9^a^	0.8^ab^	0.5^ab^	0.6^ab^	0.3^b^	0.222
	*Peptostreptococcaceae*	6.5^ab^	5.6^ab^	4.3^b^	4.7^b^	7.8^a^	0.751
	*Ruminococcaceae*	8.6^ab^	5.2^b^	6.1^b^	9.5^a^	5.6^b^	0.942
	*Erysipelotrichaceae*	6.1^c^	11.8^b^	10.6^bc^	5.4^c^	18.7^a^	2.027
	*Acidaminococcaceae*	1.6^ab^	1.3^bc^	0.7^c^	2.1^a^	0.9^bc^	0.244
	*Veillonellaceae*	2.0^c^	17.9^a^	12.8^ab^	5.5^bc^	4.8^c^	2.023
Fusobacteria		21.1^a^	13.4^b^	17.1^ab^	20.2^ab^	17.3^ab^	1.705
	*Fusobacteriaceae*	21.1^a^	13.4^b^	17.1^ab^	20.2^ab^	17.3^ab^	1.705
Proteobacteria		7.1^ab^	5.5^ab^	5.8^ab^	7.1^a^	4.2^b^	0.767
	*Succinivibrionaceae*	2.4^ab^	2.8^a^	3.0^a^	2.3^ab^	0.8^b^	0.525
	*Burkholderiaceae*	4.2^ab^	2.5^b^	2.8^b^	4.6^a^	3.3^ab^	0.422

1 *CON, Poultry by-product meal control; GBD, Garbanzo bean; GLD, Green lentil; PFD, Peanut flour; DYD, dried yeast*.

2 *SEM, Standard error of the mean*.

a−c *Means within a row with different superscript letters are different (P < 0.05)*.

The microbial composition at the family level is shown in Table 9, identifying over 30 different families. The relative abundance at the family level varied among different treatments. The most abundant families included *Fusobacteriaceae, Lachnospiraceae, Bacteroidaceae, Erysipelotrichaceae*, and *Veillonellaceae*. At the genus level, an increased relative abundance of the genus *Megamonas* was observed in dogs fed GBD and GLD compared to the remaining treatment groups. Additionally, dogs fed DYD had higher relative abundances of several genera belonging to the Frimicutes phylum compared with dogs fed the remaining dietary treatments, including *Clostridium Sensu Stricto 1, Ruminococcus Gauvreauii Group, Romboutsia, Erysipelatoclostridium*, and *Erysipelotrichaceae UCG-003*. However, due to the complexity of the hindgut microbial composition, the changes in microbial diversity for each dietary treatment were also determined.

**Table 9:** Relative abundance of bacterial genera of dogs fed diets containing legumes or yeast as the primary protein source.

		**Dietary treatment**^**1**^					
**Phylum**	**Genus**	**CON**	**GBD**	**GLD**	**PFD**	**DYD**	**SEM^**2**^**
Actinobacteria	*Collinsella*	0.4^ab^	0.8^a^	0.2^b^	0.4^ab^	0.2^b^	0.133
Bacteroidetes	*Alloprevotella*	3.3^a^	1.6^bc^	2.3^ab^	3.1^a^	0.5^c^	0.422
	*Prevotellaceae Ga6A1 Group*	2.0^a^	1.1^bc^	0.2^c^	1.5^ab^	0.8^bc^	0.257
Firmicutes	*Clostridium Sensu Stricto 1*	0.2^b^	0.2^b^	0.1^b^	0.4^b^	3.5^a^	0.506
	*Ruminococcus Gauvreauii Group*	0.1^b^	0.3^b^	0.3^b^	0.1^b^	1.6^a^	0.132
	*Ruminococcus Torques Group*	3.9^a^	1.5^b^	1.4^b^	3.1^ab^	1.9^b^	0.464
	*Peptococcus*	0.9^a^	0.8^ab^	0.5^ab^	0.6^ab^	0.3^b^	0.222
	*Romboutsia*	1.2^b^	1.7^b^	1.5^b^	1.1^b^	3.3^a^	0.343
	*Butyricicoccus*	0.1^b^	0.7^a^	0.6^a^	0.2^ab^	0.1^b^	0.109
	*Faecalibacterium*	6.1^ab^	2.9^c^	3.9^bc^	7.3^a^	4.7^abc^	0.812
	*Negativibacillus*	0.5^a^	0.1^bc^	0.1^bc^	0.5^ab^	0^c^	0.117
	*Erysipelatoclostridium*	0.4^b^	0.0^b^	0.2^b^	0.3^b^	3.4^a^	0.214
	*Erysipelotrichaceae UCG-003*	0.1^b^	0.4^b^	0.6^ab^	0.2^b^	1.4^a^	0.271
	*Faecalitalea*	0.1^b^	0^b^	0.2^b^	0.1^b^	0.6^a^	0.134
	*Phascolarctobacterium*	1.6^ab^	1.3^bc^	0.7^c^	2.1^a^	0.9^bc^	0.244
	*Megamonas*	1.9^c^	17.6^a^	12.1^ab^	5.4^bc^	4.5^c^	2.099
Fusobacteria	*Fusobacterium*	21.1^a^	13.4^b^	17.1^ab^	20.2^ab^	17.3^ab^	1.705
Proteobacteria	*Anaerobiospirillum*	2.4^a^	2.8^a^	3.0^a^	2.2^ab^	0.8^b^	0.525
	*Sutterella*	2.7^ab^	2.1^ab^	1.8^b^	3.4^a^	2.8^ab^	0.363

1 *CON, Poultry by-product meal control; GBD, Garbanzo bean; GLD, Green lentil; PFD, Peanut flour; DYD, dried yeast*.

2 *SEM, Standard error of the mean*.

a−c *Means within a row with different superscript letters are different (P < 0.05)*.

**Figure 3:**
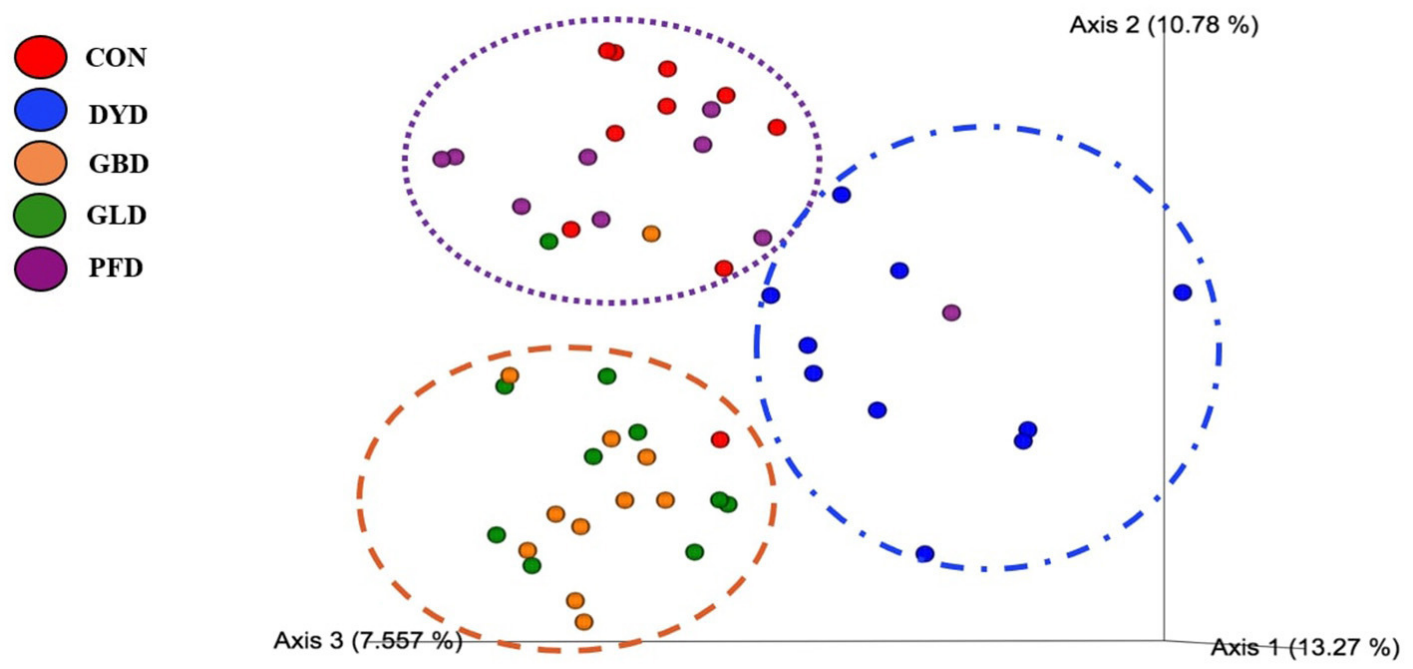
Principal coordinated plots of Bray Curtis Dissimilarity analysis of fecal microbial communities of dogs fed diets containing legumes or yeast as the primary protein source. CON, Poultry by-product meal control; GBD, Garbanzo bean; GLD, Green lentil; PFD, Peanut flour; DYD, Dried yeast.

**Figure 4:**
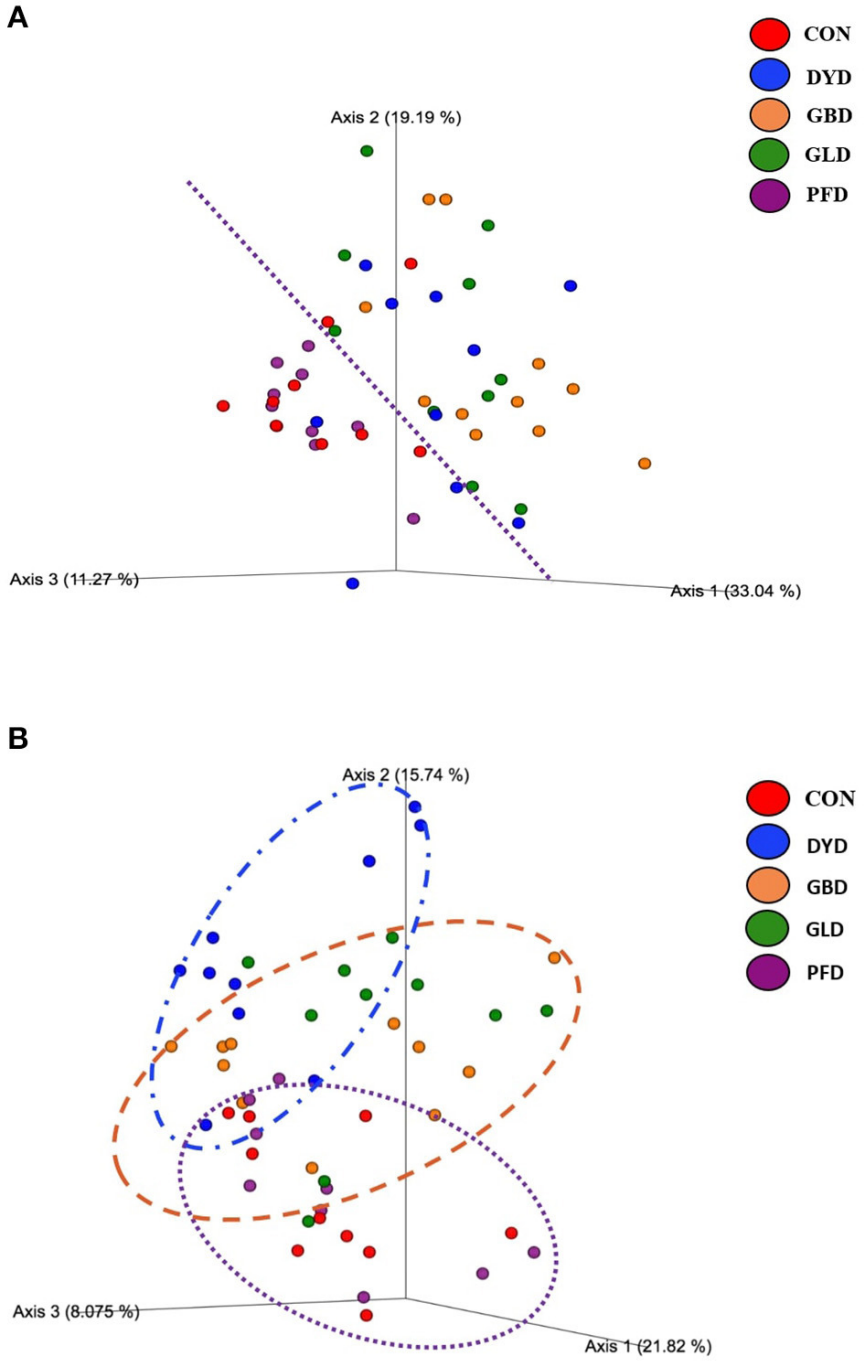
Principal coordinated plots of weighted **(A)**, and unweighted **(B)** UNIFRAC distances of fecal microbial communities of dogs fed diets containing legumes or yeast as the primary protein source. CON, Poultry by-product meal control; GBD, Garbanzo bean; GLD, Green lentil; PFD, Peanut flour; DYD, Dried yeast.

**Figure 5:**
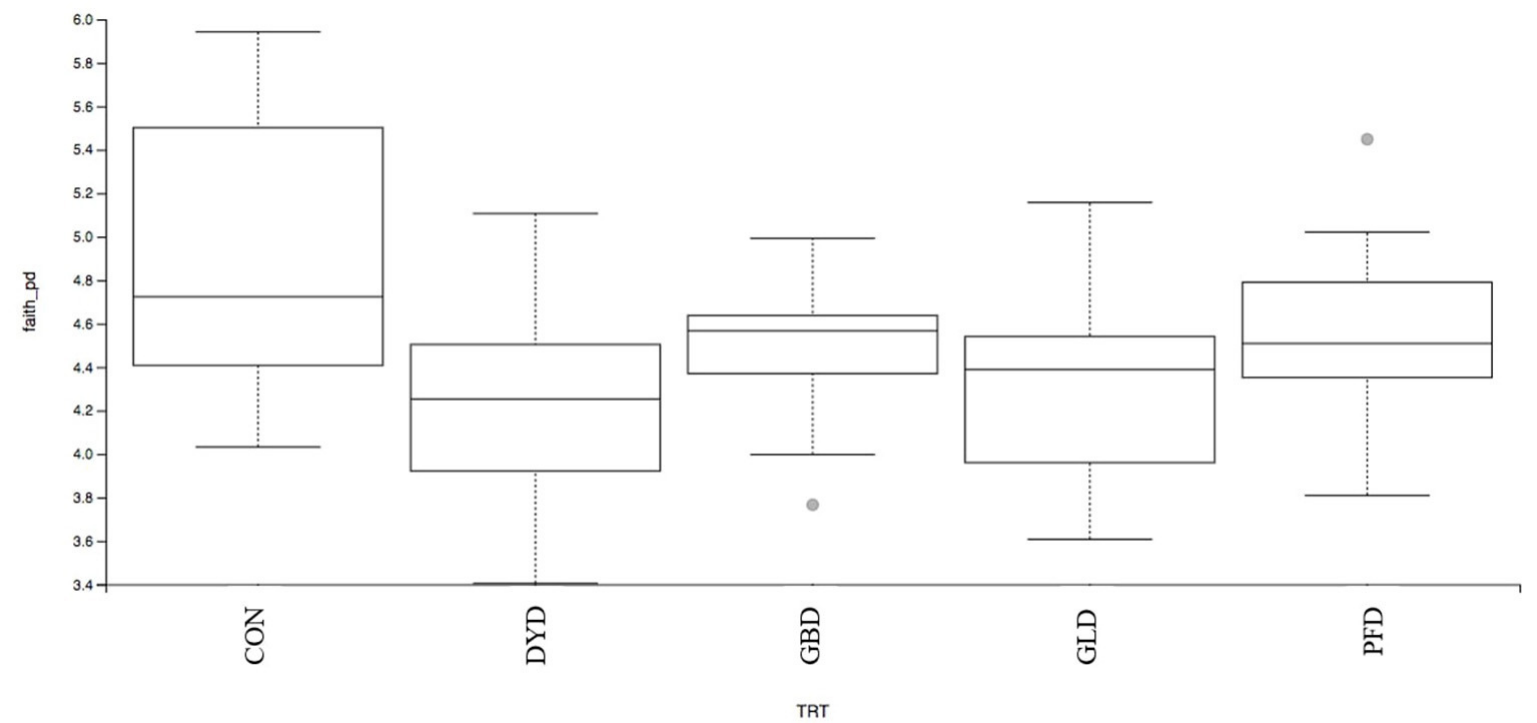
Alpha-diversity analysis of fecal microbial communities, measured by Faith's phylogenetic diversity (PD), of dogs fed diets containing legumes or yeast as the primary protein source. CON, Poultry by-product meal control; GBD, Garbanzo bean; GLD, Green lentil; PFD, Peanut flour; DYD, Dried yeast.

The β-diversity was determined based on Bray-Curtis Dissimilarity Analysis (Figure 3). Differences in microbial diversity among dietary treatments were based on a combination of *p-* and *q*-values <0.01. Dogs fed CON and PFD had similar fecal microbial communities in contrast with dogs fed DYD, GBD, or GLD. While GBD and GLD did not differ from each other, DYD resulted in a different microbial composition. The β-diversity based on weighted UniFrac analysis (Figure 4A), showed fecal microbial community abundance was similar between dogs fed CON and PFD. Additionally, dogs fed GBD and GLD had similar microbial abundance. Dogs fed DYD differed from all other dietary treatments. The β-diversity based on unweighted UniFrac analysis (Figure 4B) showed an identical pattern as the weighted UniFrac analysis. The α-diversity (Figure 5), measured using Faith's phylogenetic diversity (PD), indicated that species evenness within a sample was higher (*P* < 0.05) in dogs fed CON than dogs fed GLD.

**Figure 6:**
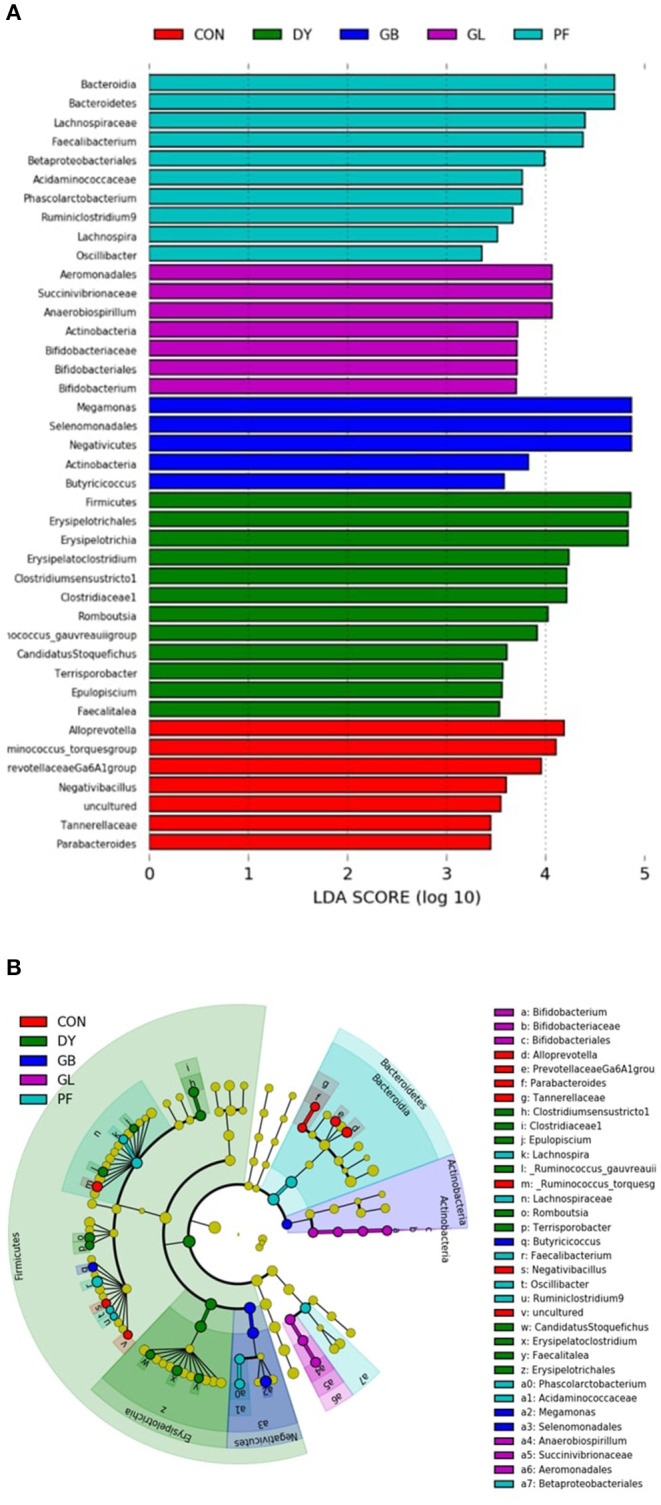
Linear discriminant analysis (LDA; **A**) effect size (LEfSe; **B**) Analysis of fecal microbiota of dogs fed legumes or yeast as the primary protein sources. CON, Poultry by-product meal control; GBD, Garbanzo bean; GLD, Green lentil; PFD, Peanut flour; DYD, Dried yeast.

The taxonomic differences were then characterized through Linear Discriminant Analysis (LDA) Effect Size (LEfSe) according to specific dietary treatments and protein sources (Figure 6). According to the LDA scores, Lachnospiraceae was a family featured in dogs fed PFD. Dogs fed GLD had the Actinobacteria phylum featured, particularly taxonomic groups related to bifidobacterium. Similar to GLD, dogs fed GBD showed Actinobacteria as a discriminant feature, along with *Megamonas*, a genus from the Firmicutes phylum, and *Negativicutes* class. Firmicutes, along with several taxonomic groups within this phylum, and Erysipelotrichales were featured in dogs fed DYD. Dogs fed CON featured taxa mostly from Firmicutes and Bacteroidetes phyla.

## Discussion

### Diet, Food Intake, and Fecal Characteristics

All 5 diets were formulated to target similar nutrient and ingredient composition, with the exception of the primary protein source, which was included at the expense of poultry by-product meal and rice. The inclusion level of the legumes and yeast ingredients varied to make the diets as isocaloric as possible. The difference in food intake (DMB) observed in dogs fed GLD and CON can be attributed to the individualized preference of the dogs or the lowered AHF content of GLD. Overall, diets were well-accepted and did not result in inadequate daily food intake or body weight loss during the study.

The large numerical differences between fecal output on an as-is basis and fecal output on a DMB are indicative of the substantial water-holding capacity of legumes. Legumes and pulses typically contain ~30% dietary fiber (2) which are responsible for the water-holding capacity of the legumes included in these diets. The water-holding capacity of legumes have been shown to increase after cooking processes (26) such as extrusion. A previous study corroborated these findings, demonstrating that the fecal moisture content of dogs fed either lentils or peas was higher than dogs fed a rice diet (27). Despite the differences in fecal output in the current study, this did not impact the fecal quality among dogs fed the different treatments.

### Maillard Reaction Products

Maillard reactions are spontaneous, non-enzymatic, browning reactions that occur between a reducing sugar and a free amino group in a protein when exposed to heat. While Maillard reactions can improve palatability, advanced stages can negatively impact protein quality (28, 29). Lysine is particularly reactive in Maillard reactions due to the presence of an epsilon amino group, reducing its availability (30). Because lysine is commonly the first or second-limiting amino acid in commercial pet foods, a reduction in lysine availability can result in decreased protein quality of the diet (31).

Hydroxymethylfurfural is a common intermediate product and a common marker of advanced stages of the Maillard reaction (12, 32). The higher concentration of HMF observed in PFD could be attributed to using a dark roasted peanut flour in the diet. While Maillard reactions that occur during the roasting process are responsible for color and flavor development of peanut flour, it can also negatively impact the protein quality of the ingredient. One study showed that as roast color darkened, the amount of available lysine present in peanut flour decreased (33). With an inclusion of peanut flour at nearly 30% in the current study, the exposure of the peanut flour to dark roasting conditions and the added exposure to extrusion temperatures could contribute to the higher concentration of HMF in the overall diet. One study estimated that a 70 kg adult human has an average daily intake of 0.28 mg HMF/kg BW^0.75^ compared with a 20 kg adult dog, fed a commercial extruded diet, which ingests 34.5 mg HMF/kg BW^0.75^ per day (12). This 122-fold difference is possibly attributed to the fact that dogs are monophagous and consistently consume higher protein commercial diets that often undergo heat processing.

The acid hydrolysis of FL causes a chemical conversion to FS, regenerated lysine, and pyridosine (34). The formation of FS is considered to be constant at about 30–34% yield (34), allowing it to be used as an indirect measure of FL. A large amount of variation has been previously reported in the FL and FS contents of canine diets based on processing or storage conditions (12, 35). Extruded, canned, and pelleted canine diets and found FL contents of 0.71, 4.46, and 0.21 g/kg (DMB), respectively (12). Additionally, the FS contents of the diets were 14.1, 21.7, and 11.5 g/kg (DMB), respectively. A different study measured the FS content of a single extruded dog diet to be 0.9 mg/g (as-is) but increased to 1.5 mg/g after 12 weeks of storage at 22.2°C or 3.2 mg/g after 12 weeks of storage at 37.8°C (35). The FL or FS contents in these previous studies are higher than the FS and FL measured in the current study, potentially due to differences in extrusion parameters or storage conditions of the diets.

The FS content of the diets was also used to calculate the concentration of reactive lysine, or the lysine available for protein synthesis (9). One of the limitations of using the furosine method to determine reactive lysine is the assumption that furosine comprises 32% of Amadori products, a value derived from the Amadori products present in milk (9, 36). Some variation in reactive lysine content has been observed in extruded canine diets, potentially due to the differences in processing parameters. A previous study has measured the reactive to total lysine content of extruded commercial canine maintenance and growth diets to be 0.85 and 0.75, respectively (37, 38). Similarly, the reactive to total lysine ratio was analyzed in commercially-available extruded, canned, and pelleted canine diets to be 0.90, 0.98, and 0.84, respectively (37, 38).

The formation of CML in foods is often used as a marker for advanced glycation end-products (AGE) that have been linked to negative health effects in humans, rats, and dogs, such as increased inflammatory and oxidative stress or insulin resistance (39–42). Therefore, CML is commonly measured in human foods to help prevent the formation of AGE (41), but CML concentrations have not been extensively measured in canine diets. One study measured the CML concentration to be 15.4 g/kg (DMB) in extruded adult canine diets. In canned adult canine diets, the CML concentration was 36.5 g/kg (12).

### Anti-nutritional Factors and Oligosaccharides

Anti-nutritional factors are protective metabolites in plants that deter consumption of the plant by birds or pests (43). Trypsin inhibitors, classified into either Kunitz or Bowman-Birk families, reduce digestion and absorption of protein in legumes through modulation of trypsin, an important protease (44, 45). However, heat processing methods have been proven to be effective in reducing or eliminating trypsin inhibitors (45, 46) increasing digestion and improving palatability of legumes (47). Extrusion has been shown to decrease trypsin inhibitor activity in extruded soybean meal diets from 2.7 mg/g to 0.6 mg/g (48). Additionally, extrusion of raw lentil flour demonstrated a 98.3% to 99.5% reduction in trypsin inhibitor activity at extrusion temperatures ranging from 140 to 180°C (49). The extrusion temperatures of the diets used in the current study were sufficient in eliminating the trypsin inhibitor activity in the final test diets and, therefore, did not negatively impact protein digestibility.

Urease is an enzyme present in many legumes that is responsible for catalyzing the hydrolysis reaction of urea to ammonia and carbon dioxide (50). Urease activity is measured through the increase in pH caused by the accumulation of ammonia (51), which can be used as an indirect marker of the presence of anti-nutritional factors. Traditionally, urease activity of 0.05 Δ pH units or less is indicative of over-processing of soybeans, while 0.25 ΔpH units or higher indicates over-processing (52, 53). However, this range corresponds to the processing of the soybean ingredient only and may not be applicable to a pet diet matrix where many ingredients have been processed prior to inclusion which explain the lack of urease activity measured in the dietary treatments used in the current study. Additionally, urease is more susceptible to heat treatment than trypsin inhibitors (54). Therefore, it would be expected that there would be negligible urease activity when trypsin inhibitors were also inactivated in the final test diets. One previous study reported similar urease activities in extruded canine diets containing traditional or defatted soybean meal or micronized, toasted, or raw soybeans (55). The urease activity of all of the dietary treatments ranged from 0 to 0.04 Δ pH units after extrusion (55). A different study analyzed the urease activity of extruded canine diets containing either 30% poultry by-product meal or soybean meal and observed slightly higher urease activity (Δ pH of 0.05 and 0.08, respectively) than that measured in the current study (56).

The inclusion of legumes in companion animal diets have been negatively associated with increased gas production and gastrointestinal discomfort (57, 58). Stemming from the rapid fermentation of α-galactooligosaccharides in the hind gut (59). These negative effects have been shown to be mitigated through heat treatment, such as extrusion (60), which may explain the lack of gastrointestinal distress observed in the dogs in the current study. However, inconsistencies in heat treatment impacting oligosaccharide concentrations have been reported (61). The raffinose and stachyose content of dried pea flour in a previous study decreased from 1.6 and 2.0%, respectively, in the raw flour to 0.8 and 1.5%, respectively, after extrusion (62). However, the oligosaccharide content in the diets from the current study did not consistently decrease throughout the stages of extrusion, as was hypothesized. A different study only noted a consistent decrease in sucrose concentration as a result of extruding black bean flour, with variable results measured for stachyose content (63). Pre-treatment of the ingredients, such as soaking or pre-cooking, could aid in decreasing the oligosaccharide content in these ingredients compared with raw ingredients (60). It has also been suggested that both cultivar and processing conditions can impact the ability of the oligosaccharides to be reduced during extrusion (61, 62).

### Apparent Total Tract Macronutrient and Energy Digestibility

Nutrient digestibility is an important factor to consider during diet formulation, as the digestibility of nutrients does not occur individually, but results from associative effects in the diet matrix (64). In the current study, all dietary treatments were considered to be well-digested by dogs, with macronutrient ATTD higher than 80% digestible. The only exception was TDF.

The macronutrient ATTD in dogs fed lentils or garbanzo beans has demonstrated variable responses in previous studies. A previous study analyzed a diet containing 69.5% lentils had apparent total tract DM, OM, CP, and fat digestibility at 74.5, 79.3, 79.9, and 89.4%, respectively (27). However, the ATTD of TDF reported for the lentil diet in this study was higher (33.4%) than the current study (26.7%). A different study measured higher ATTD of DM, OM, and CP in dogs fed a combination of 120 g/kg garbanzo beans and lentils to be 90.7, 92.3, and 92.3%, respectively (64). In dogs fed garbanzo beans and lentils, the ME content was calculated at 3.5 kcal/g (65).

Few studies have focused on evaluating the digestibility of peanut flour. One study analyzed the apparent ileal CP digestibility of a diet containing 31% peanut flour in broiler chickens and growing pigs (66). The apparent ileal CP digestibility of peanut flour was 77% when fed to broiler chickens and 79% when fed to pigs (66). Similarly, in a separate study, broiler chickens fed either 100 or 200 g/kg peanut flour meal had DM ATTD of 72 and 70%, respectively (67). In that study, the ME content of broiler chickens fed 100 or 200 g/kg peanut flour meal were estimated to be 3.77 and 3.91 kcal/g, respectively (67). As mentioned previously, the lowered CP digestibility could be a result of the roasting process of the peanuts which can damage lysine making it unavailable for protein synthesis. In one of the few studies available that evaluated peanut flour in canine diets, dogs were fed a diet containing 7% peanut flour and reported true nitrogen digestibility to be 93% compared with casein (98%), defatted whole egg powder (96%), dried egg albumin (84%), and dried beef muscle (98%) (68).

The digestibility of DYD is consistent with previous studies that evaluated various yeast products in dogs. The effects of a live yeast supplement on macronutrient ATTD in young Beagles was previously analyzed (6). The ATTD of DM (68.4%), ash (23.8%), and CP (66.5%) reported were lower than the digestibility measurements in the current study (6). The ATTD of crude fat (87.7%) measured in that study was similar to the AHF digestibility in the current study (87.9%). However, differences in fat extraction methods should be taken into account when comparing these results as crude fat often underestimates total lipid content and while creating more variability (69). Another study analyzed the effects of supplementing a *Saccharomyces cerevisiae* fermentation product on macronutrient ATTD in adult dogs (7). The apparent total DM, OM, and CP digestibilities of dogs supplemented with 125 mg/d were similar to the current study at 87.0, 89.0, and 87.7%, respectively. Dogs supplemented with 500 mg/d of the fermented yeast product had apparent total DM, OM, and CP digestibilities of 86.5, 88.6, and 86.8%, respectively (7). Lastly, a study measured the macronutrient ATTD of dogs fed 15% brewer's yeast, 15% autolyzed sugarcane yeast, or 15% integral sugarcane yeast (70). The dogs fed 15% brewer's yeast had apparent DM, OM, CP, and AHF digestibilities of 82, 86, 86, and 88%, respectively. Similarly, the DM, OM, CP, and AHF digestibilities of dogs fed integral sugarcane yeast were 82, 86, 83, and 86%, respectively, while the autolyzed sugarcane yeast was slightly lower at 79, 84, 80, and 84%, respectively (70).

### Fecal Fermentative End-Products

The production of SCFA is a result of dietary fiber entering the large intestine and being degraded by saccharolytic bacteria (71). In the current study, the fecal concentrations of SCFA from dogs fed the legume- and yeast-based diets are indicative of increased saccharolytic fermentation compared with CON. It has been reported the total SCFA fecal concentrations of dogs fed a diet containing 15% garbanzo beans to be 163 μmol/g compared with a diet containing 15% pea flour (179 μmol/g) and a commercial diet (182 μmol/g) (72). Acetate, propionate, and butyrate concentrations for the 15% garbanzo bean diet were 68, 47 g, and 15.2 μmol/g, respectively (72). The lower fecal SCFA concentrations reported compared with the current study could be due to the lower inclusion of the garbanzo beans in their diet, resulting in less saccharolytic fermentation in the hindgut.

Acetate is typically the most abundant SCFA produced (73), which is reflected in the acetate concentrations measured for all five dietary treatments in the current study. Fecal concentrations of butyrate in dogs fed DYD more than doubled that of dogs fed the remaining diets, potentially due to the β-glucan content of yeast. Previous studies did not observe the same pattern in butyrate concentrations of dogs supplemented with 125, 250, or 500 mg/d of a fermented yeast product (48.2, 35.6, and 42.8 μmol/g (DMB), respectively) compared with dogs not supplemented with yeast (38.6 μmol/g) (7). The differences may be due to the use of a fermented yeast product rather than whole-body dried yeast, as well as the lower dietary inclusion of the yeast product compared to the level of dried yeast in the current study.

Proteolytic fermentation takes place mainly in the distal large intestine, with BCFA as the primary end-products (74). Few studies are available evaluating fecal BCFA concentrations in dogs fed legume-based sources. However, similar total fecal BCFA concentrations as the current study in dogs fed 15% garbanzo beans at 13.1 μmol/g comprised of 3.8 μmol/g isobutyrate, 7.8 μmol/g isovalerate, and 1.5 μmol/g valerate have been reported (72).

### Fecal Microbiota

It has been well-established there is a definitive link between the metabolic roles of the gastrointestinal microbiota and canine health (75). Dietary modulation of the gastrointestinal microbiota can impact gut health and physiology, host immunity, and alter metabolic pathways (76). Previous studies have exemplified the use of legumes and yeast to modify the gastrointestinal microbiome.

In dogs fed 15% chickpea or peas, Firmicutes, Bacteroidetes, and Fusobacterium were the most abundant phyla represented (72), which corroborates the findings in the current study. At the genus level, dogs fed diets containing chickpeas had a lower relative abundance of *Prevotella, Alloprevotella*, and *Sutterella* than dogs fed a raw meat diet (72). In that same study, dogs fed a pea-based diet had a higher relative abundance of *Megamonas* compared to dogs fed a raw meat diet. A similar pattern was observed in the current study, with dogs fed GLD and GBD having higher relative abundances of *Megamonas* than dogs fed the remaining treatments. In addition to being a major propionate producer, *Megamonas* expresses α-galactosidase, a key enzyme in the degradation of galacto-oligosaccharides (77). Legume plants, including oilseeds like soybeans and pulses, contain galacto-oligosaccharides (i.e., raffinose, stachyose, and verbascose). These can be hydrolyzed to D-galactose and sucrose by the enzyme α-galactosidase, an enzyme not found in the canine digestive tract. Therefore, the increased relative abundance of *Megamonas* demonstrates an adaptation of the gut microbiota of dogs fed the GLD and GBD treatments to the consumption of oligosaccharides.

One study examined the influence of 25% navy beans on the canine gut microbiota using pyrosequencing of 16S rRNA and determined no changes in relative abundances at the phylum or family level compared with a negative control (a meat and bone meal and rice diet with 0% inclusion of navy bean powder) (78). In mice fed graded levels of lentils (5, 10, 20%), the relative abundance of Firmicutes accounted for 50% of the total phyla for mice fed 10 and 20% lentils but lower (42%) for mice fed 5% lentils compared with the basal diet, indicating the change may be dose-dependent (79). Increased relative abundance of *Bifidobacterium* have been reported when a bean extract diet was fed to mice (80). In this study, Actinobacteria and *Bifidobacteriaceae* were generally increased in dogs fed GLD and GBD in contrast with other treatments groups.

Previous studies have demonstrated that live *Saccharomyces cerevisiae* supplementation in dogs has decreased pathogenic bacteria, such as *Escherichia coli* (6). At the phylum level, dogs supplemented with a yeast fermentative product had a higher relative abundance of Actinobacteria and Firmicutes (7). Additionally, the fecal microbial composition of dogs supplemented with 1.4% yeast cell wall in raw chicken or beef diets. The yeast cell wall inclusion in both chicken and beef diets resulted in an increase of *Megamonas* within the Firmicutes phyla (2.4% in beef and 3.6% in chicken) compared with the 1.7% in dogs fed the control beef diet and 0.6% in dogs fed the control chicken diet (81). Saccharolytic bacteria produce SCFA through a complex cross-feeding system within the gastrointestinal microbiota (73, 82). Firmicutes has been shown to be primarily a butyrate producer in humans (83, 84), which may be an explanation for the increased fecal butyrate concentration measured in the current study.

Although diet is the primary factor determining microbial composition in the gastrointestinal tract (85), individual variation within a population can influence the dietary effect on the gut microbiota. Factors such as life stage, sex, breed, and health status can impact how the microbiota are modulated and are important considerations (86, 87).

## Conclusions

The high inclusion of legumes and yeast in extruded diets was well-accepted by dogs throughout the study. The analyzed serum chemistry and CBC were all within normal ranges for healthy adult dogs for the duration of the study. No negative effects were observed in fecal quality and all diets were highly digestible for all macronutrients. Dogs fed the experimental diets had greater SCFA concentrations than dogs fed CON. In particular, dogs fed DYD had high butyrate concentrations. Therefore, it can be concluded that these proteins are viable novel sources that can safely be included in canine diets, with inclusion levels over 40% for garbanzo beans and green lentils, and near 30% inclusion levels for peanut flour and dry yeast.

## Data Availability Statement

The data presented in the study are deposited in the Illinois Data Bank repository, accession number; 10.13012/B2IDB-4677176_V1.

## Ethics Statement

The animal study was reviewed and approved by IACUC.

## Author Contributions

GD, JH, and MG designed the experiment. LR performed the animal trials. LR and FH performed the laboratory analyses. LR and MG performed the statistical analyses, with fecal microbiota data analyzed by BS and SR-Z. LR wrote the manuscript. All authors provided intellectual input and reviewed this manuscript.

## Conflict of Interest

JH and GD are employed by ADM, company that supported this research. The remaining authors declare that the research was conducted in the absence of any commercial or financial relationships that could be construed as a potential conflict of interest.
