# Supplementary material for: Use of Legumes and Yeast as Novel Dietary Protein Sources in Extruded Canine Diets
Source: Front Vet Sci. 2021 Jun 4;8:667642. doi: 10.3389/fvets.2021.667642 (PMC8212060; doi:10.3389/fvets.2021.667642)
Supplement: Supplementary file 1 [file Table_1.docx]

| **Table 1.** Average Wenger X-115 single screw extruder processing conditions for canine diets containing legumes or yeast | | | | | |
| --- | --- | --- | --- | --- | --- |
|  | Dietary treatment^1^ | | | | |
| Measurement | CON | GBD | GLD | PFD | DYD |
| ***Raw material*** |  |  |  |  |  |
| Dry recipe density, kgs/m^3^ | 495.0 | 461.0 | 539.0 | 518.0 | 606.0 |
| Dry recipe rate, kg/hr | 493.0 | 506.0 | 496.0 | 492.0 | 502.0 |
| Feeder speed, rpm | 50.3 | 53.3 | 49.2 | 52.2 | 45.5 |
| ***Preconditioner*** |  |  |  |  |  |
| Mixing intensity, % | 30.0 | 30.0 | 30.0 | 30.0 | 30.0 |
| Large side speed, rpm | 263.0 | 263.0 | 263.0 | 263.0 | 263.0 |
| Small side speed, rpm | 377.0 | 377.0 | 377.0 | 377.0 | 377.0 |
| Cylinder steam, kg/hr | 35.1 | 39.9 | 40.2 | 39.9 | 25.2 |
| Cylinder water, kg/hr | 84.7 | 90.0 | 99.8 | 74.0 | 111.1 |
| Cylinder discharge temp, ^o^C | 73.0 | 78.0 | 77.0 | 81.0 | 61.0 |
| ***Extruder*** |  |  |  |  |  |
| Speed, rpm | 350.0 | 385.0 | 385.0 | 385.0 | 350.0 |
| Motor load, % | 57.1 | 52.3 | 59.1 | 56.3 | 53.0 |
| Motor power, kW | 24.1 | 22.1 | 28.2 | 25.5 | 21.2 |
| Knife speed, rpm | 1,200.0 | 1,200.0 | 999.0 | 999.0 | 1,201.0 |
| Zone 1 temp, ^o^C | 70.0 | 90.0 | 90.0 | 90.0 | 90.0 |
| Zone 2 temp, ^o^C | 80.0 | 95.0 | 95.0 | 95.0 | 95.0 |
| Zone 3 temp, ^o^C | 85.0 | 100.0 | 100.0 | 100.0 | 100.0 |
| Zone 4 temp, ^o^C | 90.0 | 105.0 | 105.0 | 105.0 | 105.0 |
| Zone 5 temp, ^o^C | 90.0 | 110.0 | 110.0 | 110.0 | 110.0 |
| Conehead pressure, KPA | 2,531.0 | 4,190.0 | 2,905.0 | 2,551.0 | 1,993.0 |
| Specific mechanical energy | 48.9 | 43.7 | 56.8 | 51.8 | 42.3 |
| ***Dryer*** |  |  |  |  |  |
| Zone 1 temp, ^o^C | 116.0 | 111.0 | 115.0 | 117.0 | 116.0 |
| Zone 2 temp, ^o^C | 56.0 | 58.0 | 60.0 | 61.0 | 63.0 |
| Zone 3 temp, ^o^C | 84.0 | 77.0 | 78.0 | 78.0 | 80.0 |
| Retention time - pass 1, min | 23.0 | 23.0 | 23.0 | 23.0 | 23.0 |
| Retention time - pass 2, min | 10.0 | 10.0 | 10.0 | 10.0 | 10.0 |
| Exhaust 1 temp, ^o^C | 72.0 | 64.0 | 67.0 | 67.0 | 69.0 |
| ***Final product*** |  |  |  |  |  |
| Extruder discharge density | 411.0 | 425.0 | 407.0 | 407.0 | 407.0 |

^1^CON = Poultry by-product meal control; GBD = Garbanzo bean; GLD = Green lentil; PFD = Peanut flour; DYD = Dried yeast
